# Erratum to: Sex differences in asking for counselling and psychological support from a therapeutic center (A.P.P.A.C.)

**DOI:** 10.1186/s12991-017-0129-3

**Published:** 2017-02-06

**Authors:** Anastasia Karkanis, John Kouros, Demy Kotta

**Affiliations:** 1 Association of Psychology & Psychiatry for Adults & Children, Athens, Greece; 2 Hygeia Hospital, Athens, Greece

## Erratum to: Annals of General Psychiatry 2008, 7(Suppl 1):S329 DOI 10.1186/1744-859X-7-S1-S329

The authors have revised the order of the author list for this article [1]. John Kouros has been moved from 1st to 2nd and Anastasia Karkanis has been moved from 2nd to 1st.
